# Medication errors in relation to direct-acting oral anticoagulants: a qualitative study of pharmacists’ views and experiences

**DOI:** 10.1007/s11096-023-01555-3

**Published:** 2023-03-28

**Authors:** Abdulrhman Al Rowily, Nouf Aloudah, Zahraa Jalal, Mohammed Abutaleb, Mohamed Baraka, Vibhu Paudyal

**Affiliations:** 1 School of Pharmacy, Institute of Clinical Sciences, College of Medical and Dental Sciences, Sir Robert Aitken Institute for Medical Research, University of Birmingham, Birmingham, B15 2TT UK; 2 Pharmaceutical Care Department, King Fahad Military Medical Complex (KFMMC), Medical Department, Ministry of Defense, Dhahran, Saudi Arabia; 3 Department of Clinical Pharmacy, College of Pharmacy, King Saud University, Riyadh, Saudi Arabia; 4 Pharmaceutical Care Department, King Fahad Central Hospital, Jazan Health Affairs, Ministry of Health, Jazan, Saudi Arabia; 5 College of Pharmacy, Al Ain University, Al Ain, United Arab Emirates

**Keywords:** Direct-acting anticoagulants, DOAC, Factor Xa inhibitors, Medication errors, Pharmacists, Qualitative research

## Abstract

**Background:**

Despite their effectiveness and ease of use, medication errors have been reported to be highly prevalent with direct-acting oral anticoagulants (DOAC).

**Aim:**

The aim of this study was to explore views and experiences of pharmacists on contributory factors and mitigation strategies around medication errors in relation to DOAC.

**Method:**

This study used a qualitative design. Semi-structured interviews were conducted with hospital pharmacists in Saudi Arabia. The interview topic guide was developed based on previous literature and Reason's Accident Causation Model. All interviews were transcribed verbatim and MAXQDA Analytics Pro 2020 was used to thematically analyse the data (VERBI Software).

**Results:**

Twenty-three participants representing a range of experiences participated. The analysis recognised three major themes: (a) enablers and barriers faced by pharmacists in promoting safe utilisation of DOAC, such as opportunities to conduct risk assessments and offer patient counselling (b) factors related to other healthcare professionals and patients, such as opportunities for effective collaborations and patient health literacy; and (c) effective strategies to promote DOAC safety such as empowering the role of pharmacists, patient education, opportunities for risk assessments, multidisciplinary working and enforcement of clinical guidelines and enhanced roles of pharmacists.

**Conclusion:**

Pharmacists believed that enhanced education of healthcare professionals and patients, development and implementation of clinical guidelines, improvement of incident reporting systems, and multidisciplinary team working could be effective strategies to reduce DOAC-related errors. In addition, future research should utilise multifaceted interventions to reduce error prevalence.

**Supplementary Information:**

The online version contains supplementary material available at 10.1007/s11096-023-01555-3.

## Impact statements

- Numerous factors identified contribute to DOAC safety, such as lack of healthcare professionals' knowledge and education, lack of monitoring, unavailability of antidotes, and underreporting of errors.
- Empowering pharmacists and patients are key enablers that could reduce DOAC-related medication errors.
- Gaps in education, training, collaboration and implementation of multidisciplinary approach when dealing with DOAC need to be addressed.

## Introduction

Direct-acting oral anticoagulants (DOAC) are effective treatment options in nonvalvular atrial fibrillation (NVAF) and the prevention and treatment of thromboembolic conditions [1]. They offer advantages over vitamin K antagonists such as warfarin in several aspects, including the need for less intense monitoring and reduced probability of drug-drug and drug-food interactions [2, 3]. Despite their effectiveness and ease of use, medication errors with DOAC are common [4]. A recent systematic review and meta-analysis demonstrated that approximately 20% of DOAC prescriptions have at least one error [5]. DOAC are classified as high-alert medication as per the institute for safe medication practices (ISMP), with the potential to cause severe bleeding episodes or even death when overdosed [6]. Pharmacists' roles in preventing and mitigating medication errors have been previously emphasised [5–9].

Understanding contributory factors associated with medication errors is essential to promote the safe utilisation of DOAC in clinical practice [5, 6, 8]. Reason's Accident Causation model offers a theoretical framework to investigate contributory factors and identify strategies for their mitigation [10]. Through awareness of contributory factors, areas of improvement for patient safety can be identified [11]. Reason's Accident Causation model distinguishes active failures, such as human mistakes and violations, from latent failures associated with system-related factors, such as lack of resources [10]. A previous study identified that lack of adequate knowledge and training of physicians and nurses about DOAC, lack of confidence in prescribing and administration and lack of access to clinical guidelines as key factors related to medication errors with DOAC [12]. However, pharmacists' perspectives on DOAC related errors and effective strategies to mitigate the contributory factors have not yet been investigated.

### Aim

The aim of this study was to explore views and experiences of pharmacists on contributory factors and mitigation strategies around medication errors in relation to DOAC.

### Ethics approval

Ethical approval was obtained from the University of Birmingham Research Ethics Committee (ERN_20-0551). In addition, ethical approvals from each of the participating hospitals were also obtained (164/2020; SP20/212/R; AFHER-IRB-2020-015). Informed consent was obtained prior to the study enrolment.

## Method

### Study design

This study used a phenomenological qualitative study design. In-depth interviews were conducted with pharmacists for data collection. The findings were reported using the consolidated criteria for reporting qualitative research (COREQ) [14]. The COREQ checklist includes thirty-two items that enable researchers to appropriately report essential aspects of qualitative research teams, methodology, setting and context, results, analysis and interpretation [14].

### Participants and setting

Using a purposive sampling technique, pharmacist participants were recruited from tertiary hospitals in three different cities in Saudi Arabia: Riyadh, Jazan and Dhahran. For the hospitals to be eligible as recruitment sites, they needed to have cardiology and internal medicine departments with at least two consultant cardiologists (i.e. larger centres). Pharmacists who had experiences in anticoagulation pharmacy stewardship programs or experience in DOAC ordering, reviewing and dispensing were included. Pharmacists with two different rankings per the Saudi commission for health specialities pharmacist classification system were included. These included: senior level consultant pharmacists who had graduated with a PhD or postgraduate-year 2 (PGY2) specialised residency program with three years of practice experience after graduation; and junior-level clinical pharmacists who had an accredited degree and experience in clinical pharmacy (e.g. Master, residency or PhD) but were yet not able to meet the eligibility criteria to be consultants.

### Data collection

Hospitals received an invitation e-mail to recruit research participants. A local collaborator invited all pharmacists in each hospital and outlined inclusion criteria. During pharmacy team meetings, collaborators also conveyed research objectives to potential participants. Those who replied to the invitations were given a participants' information sheet (PIS) and requested to sign consent forms.

A topic guide was developed based on previous literature and Reason's Accident Causation Model [8, 11, 13, 14]. The majority of the interview questions [supplementary electronic material 1] focused on eliciting participants' knowledge and experience with DOAC concerns around DOAC-related errors, availability of clinical guidelines and other sources of information in their institutions, their views about healthcare professionals' roles in improving DOAC safety, and their views around contributory factors to errors and mitigation strategies. The questions for the interview schedule were drafted by the first author (AA) and further refined by the research team based on advice from research collaborators at study locations. In addition, seven other pharmacists examined the topic guide to improve its clarity. The interviews were conducted in English (since English is commonly used in hospitals in Saudi Arabia and because many of the pharmacists were English speakers), recorded, and transcribed verbatim by an expert transcriber. Prior to the interviews, the study researcher described the purpose of the study and offered an opportunity for the participants to ask any questions.

Due to COVID-19 pandemic restrictions, semi-structured interviews were undertaken via Zoom videoconferencing platform. The study was conducted during September and November 2021. The primary researcher (AA) was supported by a second member of the research team (NA) as a note-taker during all interviews.

### Data analysis

Thematic analysis of interviews was performed using MAXQDA Analytics Pro 2020. (VERBI Software) [15]. Two authors (AA, NA) separately analysed each transcript, while a third author (VP) examined both versions for discrepancies. A discussion was had in the case of any disagreements to reach a consensus. As the semi-structured interviews progressed, transcripts and notes from each session were examined to establish initial codes and to detect emergent information.

To increase rigour and credibility, each interview was concluded with a verbal summary developed by the interviewer. This summary was discussed, modified when necessary, and finally confirmed with the interviewees to ensure their agreement with the recorded interview content. The two researchers (AA and NA) met after each interview and discussed the interview data. During the interviews, memos (such as noting an interviewee's facial expressions or reluctance to answer specific questions) and journaling were recorded utilising MAXQDA memos. Interviews were conducted until data saturation was reached. Data saturation was achieved when redundant responses to interview questions were observed without any new information.

## Results

Saturation of the data collection was achieved after 20 interviews, and three further interviews (total *n* = 23) were conducted for assurance. The median duration for the interviews was 32 min, ranging from 19 to 44 min. Characteristics of the participants are presented in Table 1. Reasons for non-response to the invitation were not recorded.

**Table 1:** Participants’ characteristics (*n* = 23)

Participants' characteristics	Pharmacists (*n* = 23)
*Gender*	
Female	9
Male	14
*Training background years of experience*	
< 5	2
5–10	15
11–15	5
16–20	1
> 20	0
Years of experience with DOAC orders (in years)*	
< 5	14
5–10	2
11–15	4
16–20	2
> 20	1
*Specialty or department*	
Cardiology	2
Internal Medicine	14
Unspecialised	6
Others*	1
*Current job title*	
Consultant pharmacists 12	
Clinical Pharmacists 11	

^*^Others include any specialist for pharmacist: drug information

^*^Years of experience with DOAC orders (in years): Any experience with dispensing, checking, order handling or monitoring DOAC

**Fig. 1:**
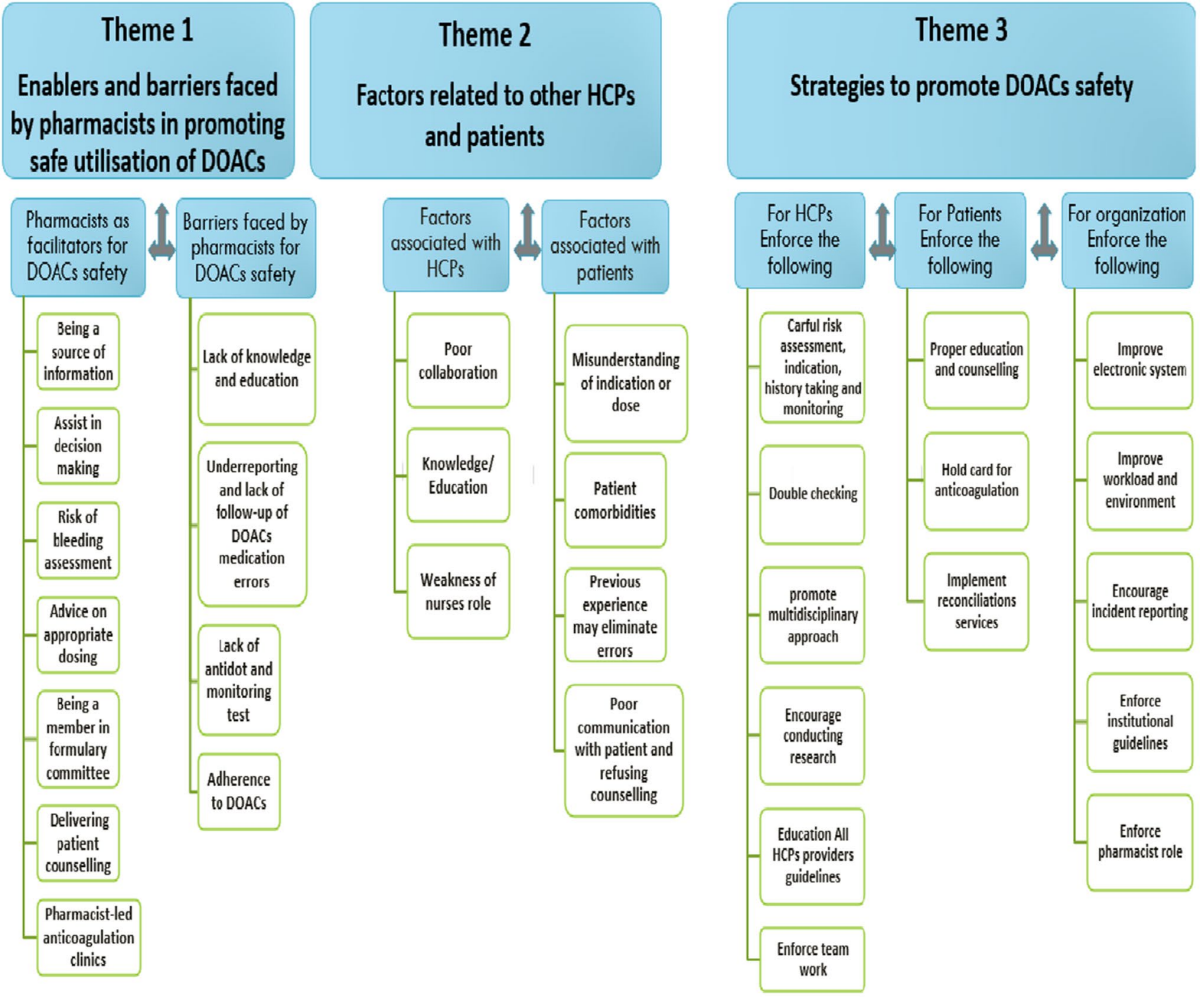
An overview of themes and subthemes HCPs: Healthcare professionals, DOAC: Direct oral anticoagulants

Three main themes were identified from the data analysis: (1) enablers and barriers faced by pharmacists in promoting the safe utilisation of DOAC; (2) factors related to other healthcare professionals (HCPs) and the patients; (3) strategies to promote DOAC safety. Figure 1 represents an overview of the themes and subthemes.

### Theme one: Enablers and barriers faced by pharmacists in promoting the safe utilisation of DOAC

1. Pharmacists as a facilitator for DOAC safety

Participants described pharmacists acting as a source of medication information. They highlighted providing recommendations to assist other HCPs in decision-making (switching from warfarin to DOACs, other elements of prescribing, and administration of DOAC), participating in bleeding risk assessment, and leading anticoagulation clinics amongst many diverse ranges of roles described (Fig. 1).

In addition, clinical and drug information pharmacists support formulary decisions that impact the use of DOAC and their safety.

“Mainly drug information pharmacist, so I dealt with most questions related to DOAC and was involved in formulary decisions related to DOAC. Those are my main two encounters, so questions received in the drug information centre and, during discussion for formulary addition” Participant 8, pharmacist (Riyadh)

Participants further illustrated activities such as conducting appropriate risk assessments to help improve safe DOAC prescribing.

“Again, you must conduct a reasonable risk assessment based on the patient's scenario, bleeding versus thrombosis risk assessment. I think you can't prescribe DOAC safely without this assessment, which is absent in many cases. I've seen they never do it.” Participant 2, pharmacist (Dammam)

Many participants described the assessment of renal function among the roles of clinical pharmacists to support decisions around the continuation, adjustment, or discontinuation of DOAC.

“One of our roles as clinical pharmacists is to usually get questions like renal dose adjustments, when can we minimise, the dose based on creatinine clearance and when to hold and when to resume and how to dose based on indication” Participant 8, pharmacist (Riyadh)

Participants also described patient counselling as one of the crucial roles of pharmacists, especially during patients' discharge. In addition, they describe pharmacists' role in providing information on the proper use of DOAC and how to deal with missing doses.

2. Barriers faced by pharmacists in promoting the safe utilisation of DOAC

Pharmacists identified a lack of knowledge of dosing guidelines and other prescribing information related to DOAC as key barriers that might lead to errors while using these high-alert medications.

“Honestly, I'm unfamiliar with all DOAC dosing (information), unfortunately, but, in some cases, I need to be more careful, and DOAC dosing in some cases are not mentioned in the guidelines” Participant 9, pharmacist (Riyadh)

Many participants reported that effective medication error reporting, investigation, and follow-up systems were lacking. From some 'participants' point of view, factors such as lack of time, workload, being bored, or just careless attitude were common reasons for underreporting of DOAC-related safety issues.

“Regarding documenting incidents through the occurrence variance reporting system in our hospital, to be honest, I don't do it. Why didn't I do that? because I don't have time, or sometimes I feel lazy to do this, to be honest.” Participant 4, pharmacist (Riyadh)

Some pharmacists believed that, unlike warfarin, DOAC lack monitoring tools or criteria for dosing. Moreover, the availability of antidotes was another problem that hindered the safe use of DOAC.

“I am feeling high responsibilities, but it is not like warfarin. Why? Because there are no monitoring criteria for the test, unlike “INR” for warfarin” Participant 1, pharmacist (Jazan)

Lastly, participants discussed the impact of patient education on adherence to DOAC and medication errors.

### Theme two: Factors related to other healthcare professionals (HCPs) and related to the patients

1. Factors associated with other HCPs

While many described positive experiences of effective multidisciplinary working, some participants described that physicians often do not accept pharmacists' recommendations after medication order review.

“… other things, the resistance from the physician side; when we contact him, he refuses to keep this medication as discontinued, so I feel under pressure if I will review that order, maybe the nurse will give it to the patient, or if I do not review it, maybe also the patient get harm if this medication not administered to him” Participant 3, pharmacist (Riyadh)

Participants further identified that knowledge of local clinical protocols and guidelines was sometimes lacking, particularly among physicians.

“Yeah, some of the physicians do not collaborate with pharmacists, especially upon catching dosing error …They should follow the local hospital protocol and guidelines, and not any other reference” Participant 7, pharmacist (Riyadh)

Participants emphasised the importance of nurses’ roles in promoting medication safety. Education and training about identifying adverse events of these drugs were deemed necessary as nurses closely monitor the patients on DOAC.

2. Factors associated with the patients

Pharmacists discussed that DOAC-related errors could be serious or fatal if patients do not understand dosing information. In addition, there was a risk that patients continued administering two anticoagulants when switched from one to the other.

“Sometimes the patient, when we shift him from one anticoagulant to another, he continues to use both.” Participant 2, pharmacist (Riyadh)

Pharmacists identified that medical history should be clear to avoid prescribing DOAC to those who have comorbidities or contraindications to these drugs.

“Wrong patient criteria; for example, the patient has advanced liver disease and was prescribed DOAC.” Participant 2, pharmacist (Riyadh)

Many noted that patients often gain knowledge through their own experiences of use.

“Yeah, of course, if the patient were more experienced with the medication, this would minimise the risk of error. if the patient has used DOAC for a long time and he/she knows what that medication used for is and what side effects may happen.” Participant 2, pharmacist (Riyadh)

Pharmacists discussed the importance of effective communication with patients in minimising errors. However, they reported that it was difficult in some situations where pharmacists were not able to communicate effectively with patients. These included situations including patients’ mental health disorders, presence of cognition-impairing diseases in addition, to illiteracy and language barriers. Some also noted that there was often reluctance from some patients to discuss medication related issues with a pharmacist.

### Theme 3: Strategies to promote DOAC safety

Study participants reported that reviewing medications by clinical pharmacists, dose adjustment according to indication and renal function, careful assessment after taking patient history, risk assessment for bleeding tendencies, providing patient education and continuous monitoring of patients could be effective strategies to promote DOAC safety.

Furthermore, pharmacists also believed that nurses could play a major role in the prevention of errors by independently double-checking DOAC orders. They emphasised that the five rights of medication administration (the right patient, the right drug, the right dose, the right route, and the right time) should also be evaluated for DOAC users before administration.

“The nurse should perform independent double checking; it is very important, especially for this high alert medication,” Participant 2, pharmacist (Riyadh)

Participants believed that HCPs should work collaboratively in multidisciplinary teams to decide about 'patients' suitability to receive DOAC therapy.

“I think forcing the implementation of the multidisciplinary approach is the number one goal to minimise the prescribing errors.” Participant 2, pharmacist (Riyadh)

Pharmacists described that research is important to generate evidence regarding DOAC prescribing in COVID-19.

“Sometimes physicians don't have any strong evidence (to support their practices with regard to DOAC, e.g., the doctors prescribe DOAC for post-COVID patients. But they don't have any strong evidence regarding this thing.” Participant 2, pharmacist (Jazan)

Participants discussed that allowing easy identification of patients on DOAC by developing specific safety cards could allow delivery of appropriate anticoagulation care and minimise medication errors. Participants also described that medication reconciliation is another high-priority strategy that pharmacists should use to promote safety.

“I believe the reconciliation is a very high priority when it comes to catching these kinds of errors. So, if we have proper reconciliation upon 'patients' admission and, discharge, you make sure that the patient has been… (looked after to avoid errors).” Participant 8, pharmacist (Riyadh)

Interviewees identified that implementing clinical decision support systems could be an effective strategy to promote DOAC safety, not only for prescribing error reduction but also for dispensing and administration errors.

Pharmacists raised the importance of improving the working environment and decreasing their workload to allow adequate time to promote patient safety. Busy shifts were deemed to negatively affect patient safety. In addition, participants discussed the importance of encouraging HCPs to report errors associated with DOAC. They emphasised that blame free reporting culture should be promoted, and the reporting system should be simplified to save time and facilitate reporting.

Furthermore, interviewees identified that improving awareness and enforcing strict audits for institutional guidelines implementation would help HCPs during the whole medication cycle, especially if such audits could be supported by a hospital information system (HIS).

“Well, I believe that, first, there should be strict protocols and guidelines, and this should be disseminated to all care providers, so they would know what the policy and educational activities is should also be done to them so they will know what's the policy.” Participant 7, pharmacist (Riyadh)

Participants indicated that empowering clinical pharmacists to perform their daily tasks in dealing with these high-alert medications was very critical. They exemplified that pharmacists can promote safety by running anticoagulation clinics, communicating their recommendations to other healthcare providers in the team, following up patients and renal adjustment of doses based on patients' kidney function.

## Discussion

### Statement of key findings

This study investigated pharmacists' views and experiences about contributory factors and mitigation strategies relevant to DOAC medication errors. Pharmacists believed that they could play a vital role in improving DOAC safety when they were provided with appropriate training and reliable information sources. These results are in line with the findings of previous studies which have highlighted the positive impact of pharmacists' interventions in the reduction of DOAC related medication errors [8]. Pharmacists could assist in clinical decision-making, perform bleeding risk assessment, help with dose adjustment, participate in formulary decisions, and provide patient counselling in addition to running specialised anticoagulation clinics [16].

The data gathered suggests that barriers to DOAC safety included poor communication with patients, lack of knowledge and training of HCPs, underreporting of errors, and lack of follow-up of patients receiving DOAC. The study further highlighted several patient-related factors such as misunderstanding of indications and proper doses, having multiple comorbidities, lack of prior experience with DOAC increased likelihood of errors. Pharmacists suggested that careful assessment of cases during history taking, better monitoring, double-checking of orders by pharmacists, multidisciplinary management, research, improving healthcare providers' awareness regarding guidelines and enforcing multidisciplinary teamwork could improve DOAC safety. Moreover, pharmacists recommended appropriate education and counselling of patients, availability of patient cards indicating the use of DOAC, and improving clinical decision support systems. They emphasised on the need to reduce the workload for healthcare providers, encourage error reporting by HCPs and updating of clinical guidelines in order to prevent medication errors.

### Interpretation

A recent study conducted in Qatar corroborated our findings in supporting the positive role of well-trained, experienced pharmacists in promoting DOAC safety. The study authors reported that clinically experienced pharmacists (i.e. those who had Board certification or clinical involvement in DOAC prescribing or dispensing) had better awareness and experience in DOAC prescriptions management, compared to other pharmacists [17].

Our study findings are concordant with the results of previously published studies that highlighted the importance of pharmacist-led clinics or services in reducing DOAC errors [5, 18]. Moreover, our study participants revealed that the clinical pharmacist role was vital to improving patient care, especially for patients with chronic disease, comorbidities and receiving polypharmacy. Pharmacists' involvement in care with other HCPs was also emphasised by physicians and nurses in our previous study [12]. Physicians' views highlighted the pivotal role of multidisciplinary teamwork in preventing medication errors [19]. Similar to the findings in this study, other literature also suggested pharmacists face similar challenges while reporting incidents of medication errors [20]. Time limitations, heavy workload, and difficulty in dealing with the reporting system were identified by pharmacists as key barriers to error reporting in our current study and the published literature [20].

Pharmacists in our study reported that improving awareness and education for healthcare providers is vital for the identification and prevention of medication errors. Implementation of education and training programs while focusing on reflective learning might ultimately reduce medication errors [20].

### Strengths and weaknesses

This study provides an in-depth understanding of contributory factors in relation to medication errors associated with DOAC from pharmacists' perspectives. Although our findings may not be generalisable due to the data generated from a qualitative study, the involvement of participants from different regions of Saudi Arabia enhanced the transferability of the findings. The participants represented healthcare institutions with diverse staffing levels, patient populations, and variable healthcare practices. Data were collected using a topic guide designed based on the literature, researchers’ previous studies, expert review and discussion amongst the research team which enhanced the face and content validity of the data collection tool.

### Further research

Our study findings provide experience-driven insights for HCPs and policymakers regarding pharmacist views about factors that could hinder DOAC safety. Effective and theory-based interventions to improve the knowledge of healthcare professionals, and patients are needed. Multifaced interventions that combine education and technology are required. Implementation and evaluation of pharmacist prescribing, pharmacist run anticoagulation clinics and development of anticoagulation stewardship programs are likely to promote safety. Future research should investigate the efficacy of proposed DOAC risk mitigation strategies. Future research findings could help prioritise these strategies based on their measured impact on promoting DOAC safety.

## Conclusion

Pharmacist participants of this study believed that DOAC safety could be improved through the education of patients and healthcare professionals, development and implementation of clinical guidelines, and improvement of error reporting systems. Furthermore, they highlighted the need to promote multidisciplinary team working. Future research should utilise multifaceted interventions to reduce error prevalence and assess the impact of such interventions.

## Supplementary Information

Below is the link to the electronic supplementary material.

Supplementary file1 (DOCX 18 kb)
